# The Hierarchic Treatment of Marine Ecological Information from Spatial Networks of Benthic Platforms

**DOI:** 10.3390/s20061751

**Published:** 2020-03-21

**Authors:** Jacopo Aguzzi, Damianos Chatzievangelou, Marco Francescangeli, Simone Marini, Federico Bonofiglio, Joaquin del Rio, Roberto Danovaro

**Affiliations:** 1 Marine Science Institute (ICM-CSIC), 08003 Barcelona, Spain; 2 Stazione Zoologica Anton Dohrn, 80122 Naples, Italy; simone.marini@sp.ismar.cnr.it (S.M.); r.danovaro@univpm.it (R.D.); 3 Jacobs University, 28759 Bremen, Germany; d.hatzievangelou@jacobs-university.de; 4 SARTI-Remote Acquisition and Data Processing Systems Research group, Electronics Department, Universitat Politècnica de Catalunya (UPC), 08800 Barcelona, Spain; marco.francescangeli@upc.edu (M.F.); joaquin.del.rio@upc.edu (J.d.R.); 5 Institute of Marine Sciences, National Research Council of Italy (CNR), 19032 La Spezia, Italy; federico.bonofiglio@sp.ismar.cnr.it; 6 Department of Life and Environmental Sciences, Polytechnic University of Marche, 60131 Ancona, Italy

**Keywords:** cabled observatories, crawler, imaging, ecological information treatment, ecological indicators, data banking, artificial intelligence, cyber-infrastructures

## Abstract

Measuring biodiversity simultaneously in different locations, at different temporal scales, and over wide spatial scales is of strategic importance for the improvement of our understanding of the functioning of marine ecosystems and for the conservation of their biodiversity. Monitoring networks of cabled observatories, along with other docked autonomous systems (e.g., Remotely Operated Vehicles [ROVs], Autonomous Underwater Vehicles [AUVs], and crawlers), are being conceived and established at a spatial scale capable of tracking energy fluxes across benthic and pelagic compartments, as well as across geographic ecotones. At the same time, optoacoustic imaging is sustaining an unprecedented expansion in marine ecological monitoring, enabling the acquisition of new biological and environmental data at an appropriate spatiotemporal scale. At this stage, one of the main problems for an effective application of these technologies is the processing, storage, and treatment of the acquired complex ecological information. Here, we provide a conceptual overview on the technological developments in the multiparametric generation, storage, and automated hierarchic treatment of biological and environmental information required to capture the spatiotemporal complexity of a marine ecosystem. In doing so, we present a pipeline of ecological data acquisition and processing in different steps and prone to automation. We also give an example of population biomass, community richness and biodiversity data computation (as indicators for ecosystem functionality) with an Internet Operated Vehicle (a mobile crawler). Finally, we discuss the software requirements for that automated data processing at the level of cyber-infrastructures with sensor calibration and control, data banking, and ingestion into large data portals.

## 1. Introduction

Earth System Science is embracing the idea that all processes on this planet are interconnected [1,2]. Ecological monitoring has been, to date, a central issue in marine sciences, since the footprint of human impacts on oceanic natural systems is growing to unparalleled historic levels [3], and industries are forcing a race for the exploitation of marine areas for which science has no available data yet [4]. In this scenario, the developments in mechatronics and robotic applications are greatly benefitting marine sciences by favoring the exploration and multidisciplinary monitoring of ecosystems [5,6]. Presently, robotic platforms are being designed for applications in the marine domain, in order to increase the effectiveness of autonomous operations and remote data acquisition and communication, as well as to overcome vessel assistance or more direct human intervention [7]. Longer autonomy in operative missions is also being implemented through the improvement of decision-making algorithms based on real-time data interpretation and of adaptive routines for the spatiotemporal exploration of monitored marine areas and for the communication of the acquired information [8]. All such capabilities rely on the augmentation of both battery duration and computational power for real-time or near real-time data processing and communication [9,10,11,12,13,14]. Such autonomous operability is increasing the inter-platform capability to communicate reciprocal location and measurements of performance and to operate cooperatively in a synchronous fashion in the same area [15,16,17,18], as successfully implemented via nature-inspired optimization approaches [19,20].

Cabled observatories can be upgraded for multidisciplinary ecological monitoring, especially when based on optoacoustic imaging applications and complex environmental data acquisition [21,22]. Optoacoustic imaging technologies (e.g., High Definition [HD], multibeam, stereo- and hyperspectral imaging, optical 3D sensors, and imaging sonars) can deliver real-time information on organisms’ presence, local abundance, as well as species composition (richness) and biodiversity, which can be related to concomitant environmental changes [23]. Imaging per se is, therefore, sustaining the revolution of the ecological monitoring of marine communities at all depths [24]. In this framework, Danovaro et al. [4] described the need for an incoming “biological revolution” based on “cameras as intelligent sensors” via Artificial Intelligence (AI) implementations in the tracking and classification of different marine species. This would enable the delivery of data on organisms and ecosystems to complement massive ecological, physical, and geochemical time series [25,26,27] in an effort to address present knowledge gaps for future marine biodiversity conservation and restoration policies. Examples of such needs, analytically presented in the recent Intergovernmental Science-Policy Platform on Biodiversity and Ecosystem Services (IPBES) and the Intergovernmental Panel on Climate Change (IPCC) reports, include ocean biogeochemistry (e.g., carbon cycle, primary production, carbon burial, etc.) and the effect of the climate and hazards on biological systems from the individual to community level (e.g., ecosystem capacity and limits of biological adaptation) [28,29]. 

Rountree et al. [30] identified different factors that limit the applicability of the video-tracking of fauna behavior, habitat resource utilization, and ecological interactions in cabled observatories, with limited spatial coverage being the principal constraining factor. In such cases, the collected information may not be fully representative of the reality it wants to describe, leading to potential problems in the form of biased interpretations of data. Statistically sound pre-planning, such as the random acquisition of information over an appropriate temporal and spatial scale, can address this issue [31] and should be more common in the marine domain. The issue of the poor representation utility of sampled marine data and the needed remedies were highlighted in [32]. Such a spatial limitation could be mitigated by deploying standardized modular units across wide bathymetric ranges and over different geographic regions. 

**Figure 1:**
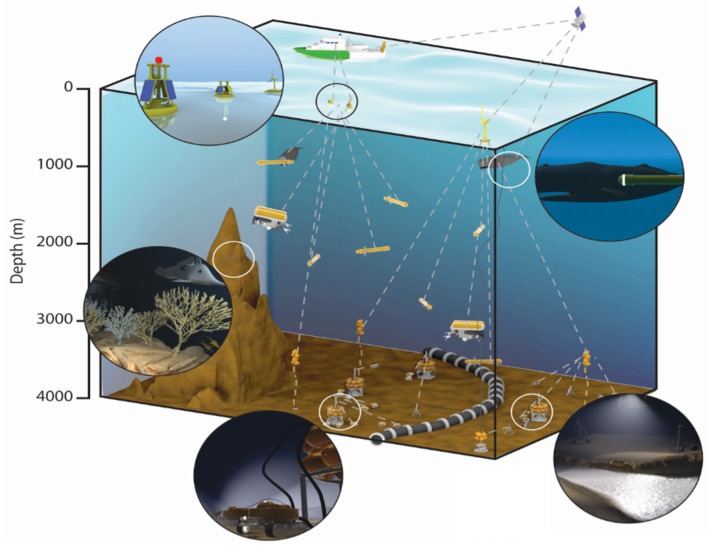
Scheme of a multidisciplinary robotized network of cabled observatories and docked mobile platforms (crawlers, Remotely Operated Vehicles [ROVs] and Autonomous Underwater Vehicles [AUVs], and benthic moorings) branching off from telecommunication cables for powering and data transfer, along with an enlarged vision of platforms and animals bearing data loggers (i.e., animal-borne technologies) in different seabed environments. These areas can become robotized marine laboratories for real-time observations, also able to gather data from the water column, as the observatory system is routinely inspected by research vessels and their assisting technologies, as well as at the sea surface by buoys and satellite teledetection.

Recently, Aguzzi et al. [23] described how fixed and mobile platforms are being organized in monitoring networks at a spatial scale capable of tracking energy fluxes across benthic and pelagic compartments, as well as across ecotones. These could represent the birth of robotized multidisciplinary ecological laboratories (i.e., where in situ manipulations can occur), deployable at virtually any depth of the continual margin and in abyssal plains, thanks to cooperation with various industries (Figure 1). Highly integrated benthopelagic monitoring providing seafloor and water column flux data (i.e., superficial and vertical profiling buoys, including neutrino telescopes) could enforce a holistic strategy in cooperation with satellites and less frequent vessel-assisted surveys. This coupled measurement would link sea surface processes (e.g., primary productivity) to carbon fluxes input at the seabed in real-time [33]. When biological data are acquired over consecutive years at a high frequency from all points within a spatial network, indicators like species density, biomass, and the resulting biodiversity can be calculated, and their status changes can be tracked according to concomitant environmental fluctuations [34,35]. In addition, relevant data on behavioral interactions among species (i.e., food web architectures) and ecological changes due to anthropic impacts can also be extracted [23]. Moreover, benthic network assets can be tuned to receive information from animal-borne technologies, delivering data on the environmental variability experienced by individuals during their displacements (i.e., data loggers storing oceanographic information of crossed seascapes [36,37]). 

As a result of this technological development, marine ecology is gradually filling up some knowledge gaps on species life traits and ecosystem functionality dynamics, which are of relevance for the conservation and management of marine ecosystems (Table 1). 

## 2. Objectives

To date, the ecological multiparametric information acquired by cabled observatories and their networks is still poorly representative of the true scale of ecological phenomena it wants to describe. That is, information collection is yet not organized in a way that properly represents the structural complexity of the marine ecosystem. This risks reducing the value of such data in terms of the true ecosystem state and dynamics. Therefore, there is clear urgency to indicate a roadmap in the handling of the increasingly automatized acquisition and treatment of marine data along the envisaged growth of robotized monitoring networks. Here, we provide a conceptual overview on the technological developments in the generation, storage, and automated hierarchic treatment of such information, such that the spatiotemporal structural complexity of a marine ecosystem might be better captured and current knowledge gaps regarding marine biodiversity and conservation might be filled. In doing so, we present an example of ecological data acquisition and processing along the computation of indicators for ecosystem functionality (i.e., population biomass, community richness, and biodiversity) to be used for the management of species of economic value. Finally, we discuss the software requirements for data processing, banking, and ingestion into large data portals.

## 3. A New Numerically-Sustained Marine Ecology 

The capability to remotely acquire multiparametric biological and environmental data at a high frequency (i.e., in real-time) continuously over several years is gradually allowing a transition from episodic to temporal analysis in quantitative marine ecology. These studies may obtain conclusions similar to those derived from land ecology based on high-frequency and long-lasting time series of multidisciplinary data [39,40,41]. Such complex quantitative analyses are facilitated by a data science approach for the acquisition and processing of marine big-data, including multivariate statistical tools. Those tools allow an exploratory analysis that counters the risk of significance inflation [42,43] in order to identify robust indicators and environmental drivers useful for ecological monitoring and management (i.e., a bottom up approach).

### 3.1. The Pipeline for the Computing of Ecological Indicators

**Figure 2:**
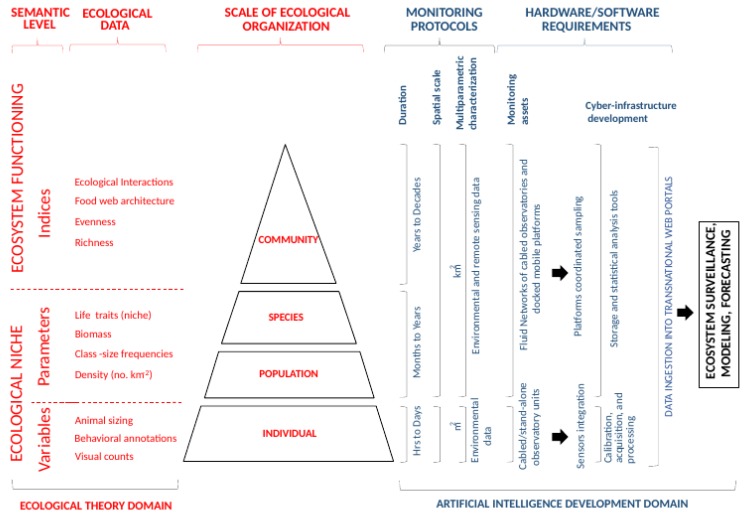
Different mechatronic hardware and software solutions (from cabled observatories to their networks, integrating docked mobile platforms) to enforce ecological monitoring at different spatiotemporal scales (from local to geographic; from days to years). As a result, data can be achieved at different levels of ecological organization (from the individual to the species and the whole community) under the form of the currently prioritized ecological indicators for management, following the strategic advice of Danovaro et al. [21]. Such spatio–temporal coordination in platform monitoring requires different levels of engineering complexity starting from the need to develop sensor calibration into a common platform environment to platforms coordinating navigation and data collection. Subsequent cyber-infrastructure development is required to store and process data, at the same time providing the tools for their online visualization and later ingestion into larger international repositories, thereby feeding ecological models and forecasts.

The rate and exchange of energy into the ecosystems determines their functionality (e.g., productivity), resulting in perceivable biodiversity, whose monitoring is of strategic importance for the management and conservation of marine ecosystems [44]. This ecological monitoring should be able to address different aspects of that ecosystem functionality and dynamics in real-time and in a continuous way, possibly over decades at adequate temporal scales via a spatially hierarchic organization of platforms (Figure 2). Accordingly, acquired multidisciplinary data from different sources may also generate different hierarchic ecological meanings (i.e., focusing on individuals and populations compared to species and communities), which are of value when dedicated to the measurement of ecosystem services and their ecological status (e.g., biomass). In this context, a pipeline to extract indicators of biodiversity and ecosystem functionality from simple input biological variables should be established as a guideline for hardware and software infrastructure development. That pipeline for ecological data treatment can be tailored trough different sequential steps that can be automated [34]:

1. The automatically acquired counts for motile megafauna species moving around a cabled observatory station, along with, for instance, animal sizing (obtained by stereo or acoustic multi-beam, time-of-flight 3D systems, or laser-scan imaging) and codified behavioral activity (e.g., scavengers, predators, etc.) are input “biological variables” that act as a first basic layer of biological information. This procedure should be done for each network platform independently.
2. A ratio between the counted animals in different observatory stations and the whole video-imaged or acoustically scanned area (by adding all fields of views together) could serve as a density estimate, which, together with rough estimates of class-size frequency distribution and total biomass could be classified as “biological parameters” in the next stage of system complexity. This procedure should be performed by pooling together data from all platforms of the monitoring network at corresponding time-lags.
3. When data on local densities are computed for all species within a list (richness), evenness can be obtained as a measure for biodiversity. Richness and evenness are ecological indicators within a third layer of sematic information since they are attributes of ecosystem functionality. At this level, ecological interactions can be calculated as descriptors for the food web architecture by multivariate statistic approaches (i.e., species clustering in a Cartesian space indicates spatiotemporal co-occurrence and may be used to highlight recurrent associations).

If feasible, all these measurements should be automatized in the form of a pipeline deployment within the observatory network. At this point, the estimation reliability would still depend on how the observatory network is set up to produce as much representative data as possible at an appropriate spatial and temporal scale.

The ecological data calculated/derived from this pipeline are meant to produce ecological models and forecasts (see Figure 2) to support biodiversity monitoring and management at an appropriate hierarchical level. That is, statistical analysis (e.g., machine learning) tools can be used to perform abundance modeling (i.e., intermediate semantic layers) of ecologically or economically important species. Similarly, one can model carbon and energy fluxes as key ecosystem functions (i.e., the top semantic layer). As the huge amount of literature on ecological modeling surpasses the goal here, we only refer to some seminal references [45,46,47,48]. However, an important note is in order. Using only classical statistical tools will likely not be enough to encompass the data complexity ensuing from the above pipeline. The field of ecological modeling is, today, quickly responding to such changes in data complexity, and substantial development in the available analysis tools is expected. Also, some modeling applications might have to live inside the marine equipment to communicate live estimates and predictions as part of the whole AI development, which could force us to think of new ways to better integrate ecological modeling in this automatic pipeline.

### 3.2. An Example with Data Form the Crawler Mobile Platform

An automatized pipeline for multiparametric data acquisition, treatment, and elaboration, consisting of consecutive and, therefore, potentially automated steps [23], is described in detail above (see Figure 2). This pipeline starts with the acquisition of biological and environmental variables (as inputs) and ends with the computing of ecological indicators (the outputs) useful for ecosystem monitoring, modeling, and forecasting. An example based on a benthic crawler is provided below (Table 2).

This crawler is a compact, mobile platform moving on caterpillars, with an umbilical cable connected to a central seafloor node providing a power supply, communication with the remote user, and data transfer [49]. It was deployed at the methane hydrate site of Barkley Canyon, off Vancouver Island (BC, Canada) as a part of the North-East Pacific Time-series Undersea Networked Experiments (NEPTUNE) cabled observatory of Ocean Networks Canada (www.oceannetworks.ca). Imaging was performed in the form of linear, constant back and forth, 20 m long imaging transects, during a period of 14 months (i.e., February 2013 to April 2014), as described in [50]. Using the standardized animal counts over a total area of 120 m^2^, the diversity indices (i.e., species richness, Shannon’s *H’*, Simpson and Fisher’s α) and biomass (i.e., wet weight per m^2^) were calculated for each month.

### 3.3. Ecosystem Analysis Challenges for Monitoring Networks

While a system like that in Figure 2 is desirable, an accurate ecosystem analysis remains challenging. For example, consider quantification of the microbial component in the water column. Here, cabled observatories and their docked platforms can hardly provide accurate estimates of microbial contributions to ecosystem functionality (i.e., the microbial loop). Imaging equipment can identify the presence of bacterial mats on the seafloor [51], but not (yet) in the water column (e.g., carbon sequestration [52]). At the same time, platforms cannot yet image marine species living within sediments (infaunal meio- or macrobenthos), leaving the biomass and biodiversity of that component largely unaccounted for [21]. Possibly, a consistent development of in situ omics-based technologies in the near future will help overcome such detection and measurement limitations, allowing the quantification and modeling of diversity and abundance of microbiome and infaunal components. Likewise, conventional imaging of pelagic zooplankton performing vertical migrations that reach the benthic layer [53] can prove testing that depends on water turbidity and the characteristics of the camera, in which case a combination of developing video (e.g., time-of flight 3D imaging [54,55] and acoustic methods such as acoustic telemetry [56], wide band technologies [57], or Acoustic Doppler Current Profiler (ADCP) backscatter [53]) would offer a broader understanding of the composition and biomass of the monitored faunal communities [58]. Here, data derived from distinct sources would have to be recompiled as described in step 2 of the pipeline for the computation of ecological indicators (see Section 3.1 above).

A similarly challenging situation occurs when automatized data collection yields information that is poorly representative of the true state of the surrounding ecosystem due to the lack of a pre-planned sampling protocol (e.g., via limited or convenient spatial coverage). This is problematic because these data might fail to produce reliable conclusions that generalize well to a broader spatial and composition scale. Here, the automatic detection of individual characteristics (e.g., animal sizing) becomes relevant to help addressing the representativeness issue. Methodological development is still needed here. However, some sources [59,60,61] point to the idea that using inverse probability weighting, based on individual characteristic information, to re-calibrate non-representative data with more accurate data on manned animal catches from pre-planned survey campaigns, might also benefit marine monitoring. Alternatively, as proposed here, a robotized monitoring network can be set-up following a pre-planned sampling protocol, when possible.

An additional difficulty is the integration of the information collected across spatially distant domains, with zones of non-instrumented areas in-between. This is typically an issue of spatial interpolation and prediction [62,63,64]. For instance, data from distant zones could be interpolated based on similar seascape information (if available) using kriging regression techniques or similar approaches. On the other hand, the standardized use of ecological indicators may help to establish a benchmark for a comparison of ecosystem functionality and productivity across areas with different monitoring platforms bearing similar sensor assets.

## 4. Cyber-Infrastructure Development

The information pipeline needs to consider a statistical approach based on an appropriate density of randomized measurements (in time and space) provided by all fixed and mobile platforms that belong to a network of observing systems. This approach provides an integrated view of the observed area within a wide range of temporal and spatial acquisition frequencies of both imaging and environmental data. This need would require a certain level of embedded capacity for data acquisition and treatment from all the involved platforms or the efficient data transfer to a central data bank for post-processing. This data bank should be implemented by automated routines for screening the data quality of each monitoring platform in the network, as well as organizing time series of variables for their real- or near real-time post processing for the production of parameter and indicator outputs. Accordingly, this data bank would be the core of a cyber-infrastructure that would manage the acquired data quality by evaluating the functioning status of all platforms and their sensors, as well as the data representation by interactive end-user interfaces for result visualization. In this sense, cyber-infrastructures should be endowed with an overall AI for data treatment, sensor integration, and data banking. 

All marine monitoring networks are getting increasingly service- and end-user oriented, with their data management cyber-infrastructures being upgraded to act as a “cognitive system” for data interpretation by humans [22,65]. Ecoinformatics provide the methodological framework to process this massive flow of information in order to extract scientifically relevant knowledge, as researchers address complex questions at scales varying from the gene to the biosphere [40,41,66,67]. Cyber-infrastructures built according to Ecoinformatics information-flow principles, on top of a hybrid-data proceeding from networked platforms, are “Virtual Research Environments” for ecological monitoring [68]. Data banks should not only be conceived of as mere repositories for multiparametric information with poor sematic value but should also provide computational tools and high-level functionality for automatically composing information workflows (i.e., from data collection at each sensor and platform to its global elaboration over the network area) [69,70,71,72]. These workflows should be capable of yielding new scientific knowledge for a diversified class of end-users spanning form scientists to citizens and policy makers. 

In order to fulfill this role, user navigation and analysis capabilities require the design of efficient web interfaces between people and data banks [73,74,75]. Ecological processes could be investigated by any end-user worldwide via those web interfaces. These interactive windows could allow us to visualize complex biological and environmental information in the form of synthetic graphic outputs, highlighting significant global change trends. Putative causes (i.e., the environmental control) and effect (ecological indicator variation) relationships could be analyzed *via* the choice of different multivariate statistics and time series analysis approaches [34,35,76,77,78] that are selected based on data quality.

### 4.1. Sensor Integration

Monitoring networks for robotic platforms require not only hardware development but also a concomitant suitable software architecture for sensor and platform control, as well as for data communication, processing, storage, and visualization [79]. The integration of sensors into marine platforms is always a challenging task when measurements have to be done in the ocean due to the variety of hardware designs and the settings of both types of components. A solution to control component interactions at different hierarchical levels of organization relies on the use of underwater “Internet of Things” approaches: the combination of sensors into platforms as physical systems upon the standardization of their interoperability, as well as the development of software. Unfortunately, we currently lack a marine internet due to limitations in platform communication for the rapid absorption of light and radio waves in seawater. 

To date, connecting any type of USB drive into a computer is a very easy “plug’n’play” operation, independent from the computer or operating system used. Ocean sensors will eventually also acquire such a degree of interoperability, once instrument and platform manufacturers all agree on a set of standards to be used. To achieve a “plug’n’play” integration of sensors into observation platforms, the host platform controller must be able to [80]:

- Detect a new sensor when it is close to a monitoring peripheral unit without human intervention (i.e., Detection);
- Obtain unambiguous description of the sensor via the transmission of metadata (ID, model, etc.) and all required information to register it to an existing sensor web server (i.e., Identification);
- Establish communication between the platform and sensor to automatically adapt the operation settings (e.g., activate a specific acquisition channel, set the sampling rate, etc.; i.e. Configuration);
- Retrieve preliminary data in order to query the sensor about the required interface asset in order to parse, process, and store the data (Simple Measurement Operations);
- Manage data streaming into the archiving server (i.e., Data Ingestion);
- Set the “plug’n’play” mechanism aimed at reducing power and computational costs and lower the bandwidth usage (i.e., Resource-Constrained).

Moreover, a critical step is the registration of sensors into existing Web services, which requires a considerable amount of metadata on their characteristics and performance status, organized and structured in a coherent way with measured parameters. Furthermore, the meaning of these metadata has to be made explicit and understandable by machines; thus, controlled vocabularies containing formal definitions, need to be used [81]. The American Ocean Observatory Initiative (OOI; https://ooinet.oceanobservatories.org/ [82]) and the European Multidisciplinary Seafloor and water column Observations (EMSO; http://www.emso-eu.org/site/) are moving in this direction. In Europe, real-time mooring using generic instrumentation, the Instrumented Interface Module (MII), and the ALBATROSS mooring line (Autonomous Line with a Broad Acoustic Transmission for Research in Oceanography and Sea Sciences) have been developed based on the Esonet NoE principle. This pioneering EMSO initiative relies on collecting all the mooring line data through an inductive communication link every thirty minutes. Data are stored on an embedded PC. Daily data files are sent to the MII through an acoustic link. ALBATROSS and MII data are sent in real time to the shore via an electro-optical cable. Cabled observatories of European seas are now evolving to provide long-term, high-resolution, and real-time deep ocean time series through a standard configuration: Each node is being equipped with an EMSO Generic Instrument Module (EGIM), incorporating standardized sensors to measure Ocean Essential Variables (EOVs; i.e., temperature, conductivity, pressure, dissolved oxygen, turbidity, ocean currents, and passive acoustics). HD cameras will be added in the near future for ecological monitoring purposes, allowing the gathering of information on relevant EOVs for megafauna related to the abundance and distribution of “Fish”, “Marine turtles, birds and mammals”, and “invertebrates”. In the medium- and long-term, the use of standards will lead to more robust and cost-efficient systems. Presently, the platform’s acoustic communication capability and its adaptive learning ability is being brought forward with different multidisciplinary hardware and software solutions in the framework of international research actions (Table 3).

### 4.2. Automated Video-Imaging 

The development of automation in image treatments for animal tracking, classification, and counting, as well as the extraction of morphological features (e.g., size and color patterning) is a pivotal aspect for the monitoring efficiency of a robotized network of fixed and mobile platforms. The software implementation sustaining the artificial computing intelligence of each unit would transform cameras into efficient sensor equivalents to any other environmental measuring device [34]. 

The general customization of automated image processing is an open issue in the scientific community of pattern analysis [83,84], and the machine learning approaches used must take into account differences in the acquisition platform, the specific location, the hardware imaging settings, and, most importantly, the wide differences of the targeted species [85]. Firstly, the huge variety of marine fauna shapes and sizes are the most relevant factors affecting the performance of shape-centered recognition and classification. Then, artificial lighting systems for illuminating the scene may complicate the identification of animals for the strong light absorption occurring in the sea water and for the scattering effects caused by the presence of suspended particulates, as in the case of turbidity and for the behavioral effects on biota. 

The relevant subjects contained in the visual data can be recognized and classified by combining computer vision and artificial intelligence methodologies, with performance comparable to that of a visual inspection operated by expert users [86]. Such a combination can be facilitated *via* a wide variety of approaches for improving the quality of images themselves [83,87,88]. Image restoration methodologies are based on a physical model of light propagation for correcting the effects of light absorption and scattering [89,90]. On the other hand, enhancement methodologies do not assume any physical model and are based only on computer vision approaches for improving light and color distribution and for reducing the hazing effects due to suspended particulates [91,92,93]. Recently developed optical 3D systems can tackle turbid waters to varying extents, depending on the monitored objects’ reflectivity and distance, with time-of-flight method sensors offering greater range while triangulating the potential for a higher resolution at short ranges [54,55].

**Figure 3:**
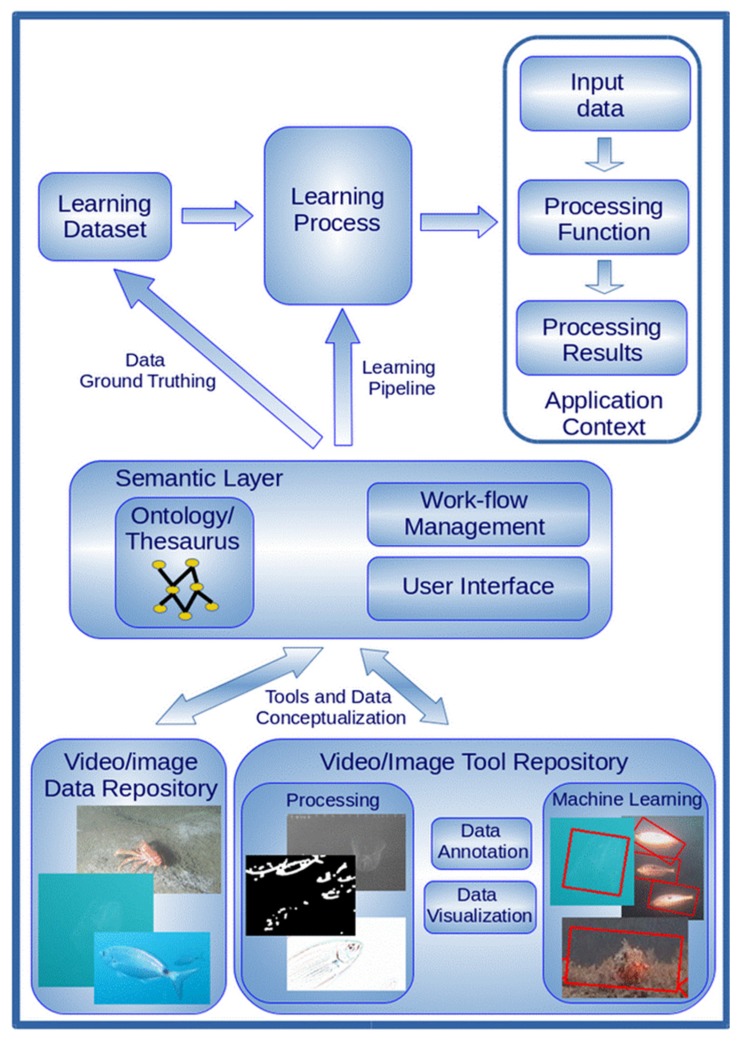
Schematic representation of different automated image processing approaches integrated into cyber-infrastructures managing semantic layer interfacing. Such an interface would allow for easy access to relevant data collections and the formal ontology/thesaurus conceptualization of the expertise needed to select and run the most appropriate computer vision and machine learning tools for content-based image analysis and processing. The work-flow management task helps scientists compose the most appropriate data analysis pipeline, while the learning process task executes the pipeline with the aim of generating a processing function capable of elaborating the input data coming from the specific application context.

Once the image improvement is carried out, animal classification can be performed by supervised machine learning approaches, where a set of examples representing all the information needed for discriminating the relevant specimens is used in learning automated algorithms for recognition and classification [14,25,94,95,96]. Advanced methodologies in computer vision and pattern recognition are emerging [97,98,99,100]. However, it is still challenging to identify the most appropriate approach for a specific application context. Moreover, in most cases, only expert computer scientists can manage the complex techniques needed for learning the appropriate classification algorithms. To overcome these limits, future cyber-infrastructures should provide advanced digital libraries not only for storing and accessing data but also the different machine learning tools capable of generating appropriate algorithms for each specific application context (Figure 3). These infrastructures should be based on high-level semantic layers (e.g., thesauruses and ontologies) for helping non expert users select the most appropriate computing approaches [101,102,103]. Infrastructures should also provide powerful hardware capabilities (e.g., computing clouds) for executing complex computational tasks (e.g., the training of neural networks and the learning of evolutionary-based algorithms) and semantic annotation tools for constructing ground-truth datasets [104,105] to be used as example datasets for the supervised machine learning approaches. All these tools and data should be freely accessible through the internet [106] and should be continuously updated as information accumulates, with the acquisition of new datasets accomplished via citizen involvement and expert supervision [35].

### 4.3. Intelligent Data Banking

**Figure 4:**
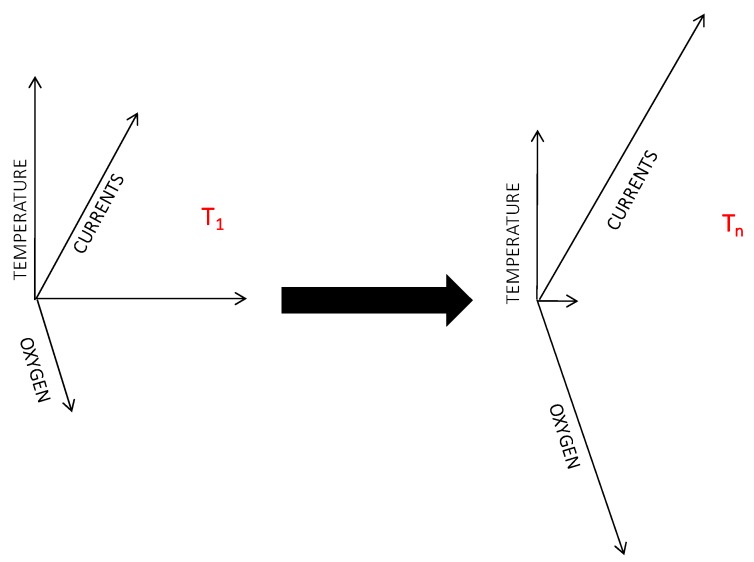
Time-lapse multiparametric data acquisition linking the detected number of individuals for a certain species at each time-lapsed (T1, Tn) image, with the concomitant variation in habitat conditioning (as represented by the status of all measured oceanographic and geochemical variables). The contextual presence of individuals within a configuration of environmental variables is an experimental approximation of the species’ ecological niche; the more diversified the group of installed sensors, the better its approximation of the niche.

We need reliable AI embedded in data banking to weight the correlations found between environmental variation and biological responses based on the autonomous analysis of data quality. Embedded data banks should include a series of routines allowing real-time or near real-time data visualization for time series plotting and subsequent statistical analysis [34,35,68]. Those graphical interfaces should be created for each ecological indicator merging all data from each platform within a network. Accordingly, some innovative and biologically-oriented developments in data banking (having cameras as “intelligent sensors” for ecological monitoring) have been proposed according to three sequential and automated stages of information processing (with a subsequent increment of the information’s semantic value), as follows (Figure 4):

(1) Structure: The synchronous real-time storage of the input row’s biological and environmental data as an incremental time series (one column per variable).
(2) Organization: The identification of a “biological event” (corresponding to the detection of an individual into an image) can be associated with a series of concomitant environmental variables (i.e., a data line) as a shapshot of the abiotic (i.e., habitat) portion of the Hutchinson niche [107].
(3) Function: The creation of a biological matrix for a species by extracting all data lines with counted individuals along with all associated environmental data. A statistically-based vision of the abiotic niche can be achieved by averaging all environmental variables at different time intervals (e.g., diel, seasonal, and annual), and tolerance levels can be assessed as the maximum and minimum values above and below which no individual is detected. Biological matrixes for all the species can then be compared together (e.g., by K-NN) defining the abiotic plus the biotic characteristics of their niches, hence providing information on their ecological interactions.

Examples of computational approaches capable of combining heterogeneous datasets into a unique explanatory/predictive model can be obtained by using evolutionary computing approaches [26,108,109]. These approaches are becoming more and more relevant in the field of knowledge discovery as they provide useful information capable of explaining the dynamics underlying the systems that generate the analyzed data.

In this scenario, archiving metadata is also a very important task to produce high-quality and intelligent data banks focused on the production of web services. These virtual facilities are available in addition to data banks to ensure the proper interoperability between data clients and providers as a key point in cyber-infrastructure design. Interesting and fast-growing solutions already exist. Oceans 2.0 from Ocean Networks Canada (ONC; http://www.oceannetworks.ca/) is presently the most advanced option for marine networks dedicated to ecological monitoring since it provides efficient and intelligent processing and analysis systems to manage the large amount of data produced from a diversified set of sensors deployed on fixed, mobile, benthic, pelagic, and even coastal platforms. The archiving system is flexible and can easily support the wide variety of data types proceeding from biological, oceanographic, and biogeochemical sensors with embedded storing, processing, and computing workflow routines that are automated. Among these scripts, automation developments centered on fauna tracking and classification for ecological indicator extraction offer the promising potential to inspire similar implementations in other growing monitoring networks, such as EMSO and OOI. 

**Figure 5:**
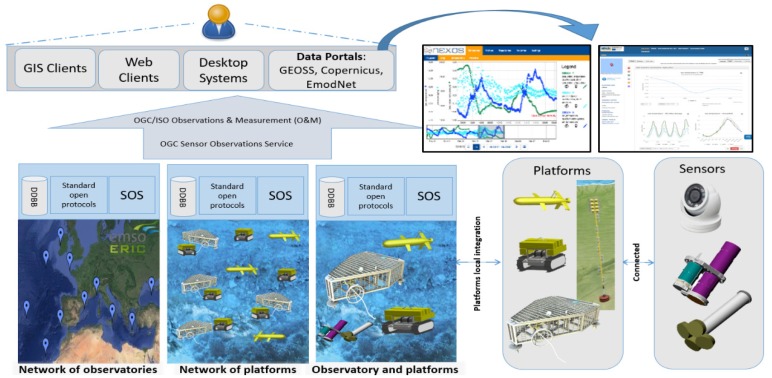
The cyber-infrastructure organization required to store, organize, and process the multiparametric information proceeding from all sensors and their platforms (as hardware environments), as organized in each network. Data from any element within a geographical group of different networks (i.e., the EMSO in the example) can be accessed through a regular web browser, since all are linked to a larger management portal, and are then available to the broader community. Benthic and pelagic (fixed and mobile) multiparametric platforms convey information to data banks and are interfaced through a Sensor Observation Service (SOS) Client. This is the same mechanisms used by portals such as the EmodNet to retrieve real-time or archived data from the platforms. This data importing from different cyber-connected repository sources allows the data to be made available to the broader community based on standardization in data acquisition, storage, and processing (i.e., Open Geospatial Consortium’s-OGC O&M). An example is presented for the OBSEA web portal that allows the access to the platform Data Bank through a regular web browser (A) and links to a larger management portal (B), connecting together different monitoring pelagic and benthic fixed and mobile multiparametric platforms.

Similar initiatives allowing users/clients to discover and retrieve available data from different pelagic and benthic sensors and their platforms and networks, all clustered together into a common cyber managing infrastructure for smart applications, are presented in Figure 5 for the OBSEA cabled video-observatory (www.obsea.es) as an example. This platform is a European Multidisciplinary Seafloor and water column Observatory (EMSO) Testing-Site and has produced multiparametric biological, oceanographic, and biogeochemical data since its deployment in 2009 [110]. Users can directly access those data from standard web browsers, but, on top of the data bank, a Sensor Observation Service (SOS; http://www.opengeospatial.org/standards/sos) is running to feed other, larger, data repositories, such as the EmodNet. A standard mechanism, which is fully automated, allows such a data bank to retrieve OBSEA multiparametric data and make them available to a broader community [80,111]. Data collected from the OBSEA observatory need to comply with Copernicus Marine Environment Monitoring Service (CMEMS) In Situ Thematic Assembly Center (INS TAC) procedures. An automatic real-time data quality control system is applied according to the manual for the real-time quality control of in-situ temperature and the salinity data of the Quality Assurance/Quality Control of Real Time Oceanographic Data2 (QARTOD, https://ioos.noaa.gov/project/qartod) project of the Integrated Ocean Observing System (IOOS, https://ioos.noaa.gov). The follow-through of the QARTOD manual for the development of these tests was made while considering the community acceptance, notability, and previous work of the IOOS. To allow proper references to OBSEA data exploitation, the datasets are registered and catalogued on Pangaea, where full data sets can be downloaded [112,113,114].

## 5. Conclusions

Human induced ecological changes are growing today at an unprecedented rate, and it is critical to adequately record and analyze these dynamics in order to plan the conservation and management strategies of important natural resources. The complexity of the natural systems under observation is daunting. Presently, marine systems are especially difficult to monitor, especially in remote or deep sections, and data retrieval in this domain has always been difficult. However, monitoring at a high temporal and spatial resolution, as well as at vast geographic scales, is needed if we want to keep pace with the fast changes in the marine environment. This observational power cannot be obtained with traditional methods.

Recently, the rapid digitalization of our instrumentation and information systems, along with hardware and software advances, promises to support the high level of environmental monitoring required to meet the needs of today’s management of marine resources. We advocate for a re-organization of these technological resources into robotized monitoring networks capable of automatically acquiring and processing complex marine data at adequate temporal and spatial scales. Those automatic networks could wildly reduce traditional monitoring costs while being more time and space effective. Such a development is, today, possible, and an overview of the technological and methodological challenges, along available solutions, further developments, and current examples of ongoing large monitoring programs, is offered.

Further developments of marine robotized monitoring networks will be inevitably based on a multi-disciplinary approach, where advances in mechatronics must meet innovations in hardware design, software programming (intelligent algorithm), and statistical methodologies in order to autonomously and flexibly answer ecologically-relevant marine questions, from the level of individual organisms up to the level of communities. In this transformation, AI-based imaging and acoustic practices are playing a central role in the production of suitable ecological indicators, requiring the background development of cyber-infrastructures for data storage, processing, sensor/platform management, and user-friendly web interfaces.

A collective effort should be devoted to finalizing the goal of implementing this highly integrative data collection approach and seizing the opportunity to conceive and set up modern marine monitoring networks, which could cost-effectively improve our knowledge and understanding of the physical, biogeochemical and biological processes in the marine environment, the response of marine ecosystems to natural and human-induced changes, and ultimately assist in the conception, design, application, and evaluation of vast-scale management and conservation policies.

## Figures and Tables

**Table 1:** Knowledge gaps existing in marine ecology, which increase according to the species distribution depth, due to difficulties in repeating the field sampling with vessel assisted technologies, at spatiotemporally representative ecological scales (adapted from [21,38]).

**Autecology**	Biomass
	Distribution ranges (bathymetric, geographic, endemism)
	Connectivity ranges of adults (passive movement by currents, active movement by locomotor activity)
	Reproduction cycles (seasonal bathymetric displacements)
	Growth cycles (growth rates and longevity)
	Sex ratio (dimorphism in color and size)
	Trophic niche (food items)
	Rhythmic mode of displacement (endobenthic, nektobenthic, and benthopelagic)
	Ethology (intra- and interspecific interactions)
	Bioturbation (burrowing, burying)
**Synecology**	Richness and Biodiversity (taxonomic and functional)
	Trophic architecture (Guilds taxonomic composition, redundancy)
	Animal-mediated benthopelagic coupling/energy transference (deep scattering layers, bioluminescence panoramas)
	Nurseries/Spawning grounds Productivity (biomasses)

**Table 2:** Monthly diversity indices and biomass estimations at the Barkley Canyon hydrate site during 2013–2014, based on the counts reported by Doya et al. [50]. For a detailed calculation of biomass see Supplementary Materials Section “Crawler Faunal Time Series Analysis”.

Indicators	Feb	Mar	Apr	May	Jun	Jul	Aug	Sep	Oct	Nov	Dec	Jan	Feb	Apr
Richness	17	17	16	13	13	16	17	17	13	14	14	14	14	13
Diversity (Shannon Index)	1.97	2.00	2.13	1.87	1.48	1.02	1.84	1.90	1.80	2.14	2.05	1.86	1.39	2.23
Diversity (Simpson Index)	0.79	0.84	0.85	0.81	0.61	0.4	0.74	0.71	0.72	0.84	0.84	0.76	0.59	0.87
Diversity (Fisher α)	3.96	3.27	3.29	2.45	2.20	2.57	3.22	3.86	2.55	2.72	2.78	2.98	2.36	2.61
Biomass (g/m^2^)	44	34	41	161	477	770	285	34	30	143	49	25	36	39

**Table 3:** Examples of EU projects related to oceanographic and coastal multiparametric monitoring, where platform and cyber-infrastructure developments could serve as a model for benthic ecology-oriented networks. Projects reproduce different operative scenarios using a variety of sensors, platforms, and sets of Open Geospatial Consortium (OGC) protocols (e.g., Sensor Web Enablement; SWE, Programmable Underwater Connector with Knowledge; PUCK (standard protocol), and Sensor Observation Service; SOS), in order to demonstrate the feasibility of the use of standards to achieve such a degree of interoperability.

Project	Acronym	Web
Optimizing and Enhancing the Integrated Atlantic Ocean Observing Systems	AtlantOS	https://www.atlantos-h2020.eu/
Bringing together Research and Industry for the Development for the Development of Glider Environmental Services	BRIDGES	www.bridges-h2020.eu
Copernicus Marine Environment Monitoring Service	CMEMS	http://marine.copernicus.eu/
European Marine Observation and Data Network	EmodNet	www.emodnet-physics.eu/Portal
European Multidisciplinary Seafloor and water-column Observatory	EMSO-ERIC	http://www.emso-eu.org/
European Global Ocean Observing System (SeaDataNet and SeaDataCloud)	EuroGOOS	www.seadatanet.org
Fixed-Point Open Ocean Observatories	FixO_3_	www.fixo3.eu
Global Earth Observation System of Systems	GEOSS	www.earthobservations.org/geoss.php
Towards a joint European research infrastructure network for coastal observatories	JERICO-NEXT	http://www.jerico-ri.eu/
NEXt generation, cost-effective, compact, multifunctional web enabled Ocean Sensor systems empowering marine, maritime and fisheries management	NEXOS	www.nexosproject.eu
Ocean Data Interoperability Platform	ODIP I/II	www.odip.eu
In Situ Chemical Mapping probes	SCHeMA	www.schema-ocean.eu
Marine sensors for the 21st Century	SenseOCEAN	www.senseocean.eu/
Sensing, monitoring and actuating on the Underwater world through a federated Research InfraStructure Extending the future internet	SUNRISE	http://fp7-sunrise.eu/
Improving and integrating European ocean observing and forecasting systems for sustainable use of the oceans	EUROSEA	https://www.eurosea.eu/
Blue-Cloud: Piloting innovative services for Marine Research and the Blue Economy	Blue-Cloud	https://cordis.europa.eu/project/id/862409

## Supplementary Materials

The following are available online at https://www.mdpi.com/1424-8220/20/6/1751/s1, **Table S1**. Data sources for the steps in biomass estimation.
