# Supplementary material for: The Hierarchic Treatment of Marine Ecological Information from Spatial Networks of Benthic Platforms
Source: Sensors (Basel). 2020 Mar 21;20(6):1751. doi: 10.3390/s20061751 (PMC7146366; doi:10.3390/s20061751)
Supplement: Supplementary file 1 [file sensors-20-01751-s001.pdf]

sensors

MDPI

Review

The Hierarchic Treatment of Marine Ecological Information from Spatial Networks of Benthic Platforms

Jacopo Aguzzi [1,2,*], Damianos Chatzievangelou [3], Marco Francescangeli [4], Simone Marini [2,5], Federico Bonofiglio [5], Joaquin del Rio [4] and Roberto Danovaro [2,6]

1 Marine Science Institute (ICM-CSIC), Paseo Maritimo de la Barceloneta, 37-49. Barcelona 08003, Spain
2 Stazione Zoologica Anton Dohrn, Naples 80122, Italy; simone.marini@sp.ismar.cnr.it (S.M.); r.danovaro@univpm.it (R.D.)
3 Jacobs University, Bremen 28759, Germany; d.hatzievangelou@jacobs-university.de
4 SARTI - Remote Acquisition and Data Processing Systems Research group, Electronics Department, Universitat Politècnica de Catalunya (UPC), Barcelona 08800, Spain; marco.francescangeli@upc.edu (M.F.); joaquin.del.rio@upc.edu (J.d.R.)
5 Institute of Marine Sciences, National Research Council of Italy (CNR), La Spezia 19032, Italy; federico.bonofiglio@sp.ismar.cnr.it
6 Department of Life and Environmental Sciences, Polytechnic University of Marche, Ancona 60131, Italy
* Correspondence: jaguzzi@icm.csic.es

Received: 19 February 2020; Accepted: 19 March 2020; Published: date

Crawler Faunal Time Series Analysis

The time series used for the estimation of the ecological indicators (i.e., diversity indices and biomass) were based on the animal counts in video transects published by Doya et al. [1]. Diversity analyses were performed with the "vegan" R package [2]. For biomass, animal densities (N/m^2) were calculated with the standardized counts over a total area of 120 m^2. Wet weights of animals were either calculated using growth curves based on animal sizes or, when sizes were not available, the published average weights, preferably from NE Pacific, were used instead. When available, data from comparable depth strata, such as the Barkley Canyon hydrates (~870 m), were preferred, while any reported differences in the vertical distribution of each species depending on sex and maturity level were also taken into account. More details for the procedure followed for each species are presented in Supplementary Table S1 below. All references are also included in the reference list in the main text.

Supplementary Table S1. Data sources for the steps in biomass estimation.

Species	Size	Weight
Licenchelys sp.	-	Average weight [3]
Microstomus pacificus	Average size [4]	Growth curve [5]
Embassichthys bathybius	Average size [4]	Growth curve [5]
Hippoglossus stenolepis	Average size [3]	Growth curve [5]
Coryphaenoides sp.	Average size [6]	Growth curve [5]
Sebastidae	Average size [7]	Growth curve [5]
Bathyagonus nigripinnis	Average size [8]	Growth curve [5]
Anoplopoma fimbria	Average size [9]	Growth curve [5]
Eptatretus stoutii	Average size [10]	Growth curve [5]
Salpidae	-	Average weight [11]
Opisthoteuthidae	-	Average weight [3]
Gonatus sp.	-	Average weight [3]
Buccinoidea	-	Average weight [3]

Ophiuroidea	-	Average weight [3]
Asteroidea	-	Average weight [3]
Zoroasteridae	-	Average weight [3]
Hippasteria sp.	-	Average weight [3]
Pannichia moseleyi	-	Average weight [3]
Paguroidea	-	Average weight [3]
Lithodes couesi	-	Average weight [3]
Small crabs	Average size [12]	Growth curve [13]
Chionnecetes tanneri	Average size [13]	Growth curve [14]
Bolinopsis infundibulum	Average size [14]	Growth curve [13]
Poralia rufescens	Average size [15]	Growth curve [15]
Solmissus sp.	Average size [15]	Growth curve [15]
Voragonema pedunculata	Average size [16]	Growth curve [16]

References

1. Doya, C.; Chatzievangelou, D.; Bahamon, N.; Purser, A.; De Leo, F.C.; Juniper, S.K.; Thomsen, L.; Aguzzi, J. Seasonal monitoring of deep-sea megabenthos in Barkley Canyon cold seep by internet operated vehicle (IOV). *PLoS ONE.* **2017**, *12(5)*, e0176917, doi: 10.1371/journal.pone.0176917.
2. Oksanen, J.; Blanchet, F.G.; Friendly, M.; Kindt, R.; Legendre, P.; McGlinn, D.; Minchin, P.R.; O'Hara, R.B; Simpson, G.L.; Solymos, P.; et al. vegan: Community Ecology Package. R package 2.5-2 2018, available online: https://CRAN.R-project.org/package=vegan (accessed on 20 March 2020).
3. Hoff, G.R.; Britt, L.L. Results of the 2010 eastern Bering Sea upper continental slope survey of groundfish and invertebrate resources. U.S. Dep. Commer., NOAA Technical Memorandum NMFS-AFSC-224; U.S. Department of Commerce: Washington, DC, USA, 2011, pp. 300.
4. Vetter, R.D.; Lynn, E.A.; Garza, M.; Costa, A.S. Depth zonation and metabolic adaptation in Dover sole, *Microstomus pacificus*, and other deep-living flatfishes: factors that affect the sole. *Mar. Biol.* **1994**, *120(1)*, 145-159, doi: 10.1007/BF00381950.
5. Garaway, C.; Arthur, R.; Measuring fish catch and consumption: Practical methods for small-scale fisheries based on length as an alternative to weight-based approaches. *Fish. Manag. Ecol.* **2019**, 00, 1-9, doi: 10.1111/fme.12409.
6. Drazen, J.C.; Yeh, J. Respiration of four species of deep-sea demersal fishes measured *in situ* in the eastern North Pacific. *Deep-Sea Res. I* **2012**, *60*, 1-6, doi: 10.1016/j.dsr.2011.09.007.
7. Haigh, R.; Schnute, J.T.. The longspine thornyhead fishery along the west coast of Vancouver Island, British Columbia, Canada: portrait of a developing fishery. *N. Am. J. Fish. Manag.* **2003**, *23(1)*, 120-140, doi: 10.1577/1548-8675(2003)023<0120:TLTFAT>2.0.CO;2.
8. Haight, R.E.; Reid, G.M.; Veemes, N. Distribution and habitats of marine fish and invertebrates in Katlian Bay, Southeastern Alaska, 1967 and 1968. U.S. Dep. Commer., U.S. Dep. Commer., AFSC Processed Report 2006-06, 2006, pp. 60.
9. Doya, C.; Aguzzi, J.; Pardo, M.; Company, J.B.; Costa, C.; Mihály, S.; Canals, M. Diel behavioral rhythms in sablefish (*Anoplopoma fimbria*) and other benthic species, as recorded by the Deep-sea cabled observatories in Barkley canyon (NEPTUNE-Canada). *J. Mar. Syst.* **2014**, *130*, 69-78, doi: 10.1016/j.jmarsys.2013.04.003.
10. Leask, K.D.; Beamish, R.J. Review of the fisheries and biology of the Pacific hagfish (Eptatretus stoutii) in British Columbia, with recommendations for biological sampling in a developmental fishery. Canadian Stock Assessment Secretariat Research Document 205; Fisheries and Oceans Canada: Ottawa, Canada, 1999, pp. 48.
11. De Blois, S. The 2017 Joint U.S. and Canada Pacific Hake Integrated Acoustic and Trawl Survey: Cruise Report SH-17-07, U.S. Dep. Commer., NOAA Processed Report NMFS-NWFSC, 2019, doi: 10.25923/fk29-dx71.
12. Tester, P.A.; Carey Jr., A.G. Instar identification and life history aspects of juvenile deepwater spider crabs, *Chionoecetes tanneri* Rathbun. *Fish. Bull.* **1986**, *84(4)*, 973-980.
13. Keller, A.A.; Buchanan, J.C.; Steiner, E.; Draper, D.; Chappell, A.; Frey, P.; Head, M.A. Size at maturity for grooved Tanner crab (*Chionoecetes tanneri*) along the US west coast (Washington to California). *Fish Oceanogr.* **2016**, *25(3)*, 292-305, doi: 10.1111/fog.12155.

14. Bailey, T.G.; Youngbluth, M.J.; Owen, G.P. Chemical composition and oxygen consumption rates of the ctenophore *Bolinopsis infundibulum* from the Gulf of Maine. *J. Plankton Res.* **1994**, *16(6)*, 673-689, doi: 10.1093/plankt/16.6.673.
15. Bailey, T.G.; Youngbluth, M.J.; Owen, G.; Chemical composition and metabolic rates of gelatinous zooplankton from midwater and benthic boundary layer environments off Cape Hatteras, North Carolina, USA. *Mar. Ecol. Prog. Ser.* **1995**, *122*, 121-134, doi: 10.3354/meps122121.
16. Bailey, T.G.; Torres, J.J.; Youngbluth, M.J.; Owen, G.P. Effect of decompression on mesopelagic gelatinous zooplankton: a comparison of *in situ* and shipboard measurements of metabolism. *Mar. Ecol. Prog. Ser.* **1994**, *113*, 13-27, doi: 10.3354/meps113013.

© 2020 by the authors. Submitted for possible open access publication under the terms and conditions of the Creative Commons Attribution (CC BY) license (http://creativecommons.org/licenses/by/4.0/).
